# A Rarity Among the Rare: Psychiatric Manifestations in a Young Woman With Stiff-Person Syndrome

**DOI:** 10.7759/cureus.26745

**Published:** 2022-07-11

**Authors:** Derek Sanak, Roberto Marticorena-Martinez, Erick Acosta

**Affiliations:** 1 College of Osteopathic Medicine, Nova Southeastern University, Fort Lauderdale, USA; 2 Psychiatry, Mount Sinai Medical Center, Miami Beach, USA; 3 Psychiatry, Florida International University, Miami, USA

**Keywords:** encephalomyelopathy, anoxic encephalopathy, autoimmune encephalopathy, antipsychotic medication, first episode psychosis, neuropsychiatry manifestations, stiff-person syndrome, psychiatry case report

## Abstract

Stiff-person syndrome (SPS) is a rare progressive neurologic disease associated with autoantibodies against glutamic acid decarboxylase, an intracellular enzyme involved in the production of gamma-aminobutyric acid.

We present a case involving a 35-year-old Haitian female who was placed under the Baker Act in the emergency department for suicidal behavior and acute psychosis. She has a history of SPS with a positive glutamic acid decarboxylase (GAD) antibody, a condition most commonly found in females between 20 and 50 years of age. The condition was managed by an outpatient neurologist using diazepam, baclofen, and monthly intravenous immunoglobulin treatments. She also has an extensive history of organic neurological conditions, including traumatic brain injury at 18 years old and COVID-19-related anoxic encephalitis that occurred in December 2020. Both psychiatric and neurological physical exams were completed. They revealed a cerebellar tremor, bilateral ptosis, poor eye contact, decreased concentration, poor insight, depressed mood, flat affect, poor judgement, delusional thoughts and a disorganized thought process with tangential speech. CT and MRI imaging of the brain showed no acute intracranial abnormalities. A quantitative titer of the GAD antibody was completed and shown to be elevated >250IU/mL. Depakote 500mg twice daily and risperidone 3mg twice daily were prescribed. The patient had progressive improvement of psychosis including delusional thoughts over the following five days and was able to be discharged with instructions to follow-up with outpatient neurology.

## Introduction

Stiff-person syndrome (SPS) is a neurological disease associated with autoantibodies that inhibit the enzyme glutamic acid decarboxylase (GAD), an intracellular enzyme that functions to metabolize glutamate into gamma-aminobutyric acid (GABA). GABA acts as the primary inhibitory neurotransmitter in the central nervous system (CNS). Due to the resulting decrease in GABA within the CNS, SPS causes progressive encephalomyelopathy that is typically characterized by symptoms of rigidity, increased reflexes, and muscle spasms in the trunk and limbs, especially the lower limbs. The prevalence of SPS is extremely rare, affecting only one to two people per million. Moreover, there have been very few reports of psychiatric disease presenting in patients with stiff-person syndrome. In this case report we describe an adult patient with a long-standing history of SPS who recently began exhibiting symptoms of intermittent psychosis over the past several months, requiring multiple hospitalizations and psychopharmacological treatment. 

## Case presentation

We present a 35-year-old Haitian female with a medical history significant for multiple organic neurological conditions including traumatic brain injury (TBI) after a fall from a balcony at 18 months old, coronavirus disease 2019 (COVID-19)-related anoxic encephalopathy, neuromyopathy, and SPS with positive GAD antibody, which was diagnosed in 2014. Because of the patient’s psychiatric state, much of her history was obtained from family. She was noted to have grown up with a learning disability following the TBI. In December 2020 she was hospitalized for COVID-19 requiring endotracheal intubation, which progressed to anoxic encephalopathy. She was then transferred to another hospital and admitted until May 2021. During this time she began to have her first-ever episode of psychiatric symptoms, including disorganized words, incoherent speech, and delusions such as large corporations trying to harm her and her family. She had no previous psychiatric diagnosis and no significant family history of any psychiatric illness. She was diagnosed with acute psychosis and medically cleared without needing psychiatric medications. She was psychologically stable until January 2022, when she was admitted to another hospital by psychiatry under the Baker Act for paranoid delusions that the Haitian president (who is deceased) wanted to kill her. She was medically cleared from that hospital after seven days and discharged with a prescription for risperidone 3mg twice daily. 

In February 2022, the patient presented to the hospital with her family after two days of worsening tachypnea and agitation. Shortly after, she was placed under a Baker Act in the emergency department after stating that she wanted to die and that a group of people involving the Haitian president was trying to harm her and her family. Family reported that for the past month the patient had intermittent periods of disorganized speech, “word salad,” and paranoid delusions multiple times per day, returning to her baseline mental status of being alert and fully oriented in between these episodes. During our consult the patient had a labile affect and poor eye contact. When asked about her upbringing she became confused and exhibited disorganized and tangential speech. She believed that she had seen the Haitian president alive in her room the previous week and that there was a group of people who wanted to harm her family and assassinate this president. She stated that the television in her hospital room was sending secret messages and that she was sending them back. “Things I watch on TV and sing out loud become true.” She initially presented calm and cooperative but became agitated after being told of the plan to admit her to inpatient psychiatry, throwing her food tray full of food and coffee across the room. We completed a neurological exam that was significant for a finger-to-nose test showing a slow, high-amplitude intention tremor consistent with a cerebellar tremor. A urine drug screen was completed and came back negative except for benzodiazepines, a normal finding given that she was prescribed diazepam 5mg twice daily. Her only other prescribed medications were risperidone 3mg twice daily, baclofen 10mg, Aspirin 81mg, and monthly intravenous immunoglobulin (IVIG) infusions by her neurologist. A quantitative titer of anti-GAD65 antibody was ordered, which was shown to be significantly elevated >250IU/mL. Her psychiatric symptoms progressively improved and she returned to her baseline mental status of being fully alert and oriented to person, place, time and situation. Over the next week after being switched from risperidone 3mg twice daily to divalproex (Depakote) 500mg delayed-release twice daily. She remained psychologically stable and was able to be discharged by the end of the week with this new medication regimen and instructions for continued outpatient follow-up. 

## Discussion

Because of the rare prevalence of SPS among the population and the known reported cases showing associations between autoimmune encephalitis and psychiatric diseases, it is important to document any incidence of psychiatric symptoms that occur in a patient with SPS. We propose a possible association between SPS and the patient’s new onset of psychiatric symptoms due to the progressive nature of the disease and the significantly elevated level of anti-GAD antibody at the time of presentation. Alternatively, in considering the close temporal relationship of anoxic encephalopathy with the onset of her first-ever psychiatric event, it also seems plausible that her psychiatric symptoms were induced by ischemic encephalopathy. The patient was diagnosed with SPS in 2014, but her first psychiatric symptoms began in May 2021 when she was diagnosed with acute psychosis during her recovery in the hospital just several months after developing anoxic encephalopathy. It is difficult to be certain whether either diagnostic theory contributed to the patient’s psychiatric condition; however, there have been reported cases showing an association of the onset of psychiatric disease, including psychosis, with autoimmune encephalitis [1] as well as encephalopathies from other etiologies including hypoxic-ischemic brain injury. With regard to the former, instances of acute and schizophreniform psychoses have been described in a number of cases involving autoantibody-associated autoimmune encephalitis (AE), including GAD65-associated AE [2]. Some of these cases require antipsychotic medications to manage [3]. This may initially mimic primary psychosis. Evidence has shown schizophrenia to be associated with lower GAD mRNA expression [4]. The hypothesis for this association describes GABA’s involvement in the dopamine hyperactivity found in schizophrenia [5]. We were able to successfully treat this patient by switching her antipsychotic medication to divalproex, which metabolizes into valproic acid, an anticonvulsant medication that is recommended for symptomatic management of psychosis patients with autoimmune encephalitis [6]. Valproic acid has commonly been used successfully to treat psychotic disorders, including bipolar disorder in a patient with SPS [7]. The medication inhibits the enzyme GABA transaminase to increase concentrations of GABA in the brain, restoring the GABA lost as a result of anti-GAD antibody inhibiting the metabolism of glutamate to GABA in the CNS of this patient.

## Conclusions

It is difficult to determine if the psychiatric symptoms that this patient experienced were due to a primary psychotic disorder versus occurring secondary to a previous medical condition. In considering the diagnosis of her symptoms occurring secondary to anti-GAD65 AE, we would expect an elevated level of anti-GAD antibodies, despite the patient receiving monthly IVIG treatments. Therefore, it was important to obtain a quantitative titer of anti-GAD65 in the patient while she was experiencing symptoms of psychosis. There is a lack of evidence-based studies regarding the treatment of psychiatric symptoms in patients with SPS. Here we have described a case in which divalproex was used successfully to treat psychosis in a patient with SPS and concurrent elevated anti-GAD65 antibodies.
